# Application of Artificial Intelligence and its Subsets in Various Stages of Knee Arthroplasty from Pre-op to Post-op: An Overview

**DOI:** 10.26502/josm.511500185

**Published:** 2025-03-11

**Authors:** Jacqueline B Anderson Enni, Devendra K Agrawal

**Affiliations:** Department of Translational Research, College of Osteopathic Medicine of the Pacific, Western University of Health Sciences, Pomona, California, USA

**Keywords:** Artificial intelligence, Deep learning, Knee arthroplasty, Knee replacement, Machine learning, Orthopedic surgery, Osteoarthritis, Total knee arthroplasty, Total knee replacement

## Abstract

Osteoarthritis is a complex and painful condition marked by joint destruction and remodeling, often resulting from overuse, aging, or genetic predisposition. Surgical arthroplasty is a widely used treatment to replace degenerated joints, relieve pain, and restore function, often caused by osteoarthritis. Artificial intelligence, including its subsets such as machine learning and deep learning, has emerged as a tool to enhance knee arthroplasty by improving diagnostic accuracy, surgical efficiency and patient outcomes. This article addresses the diverse applications of artificial intelligence across the preoperative, perioperative, and postoperative stages of knee arthroplasty, categorizing studies by their focus on patient education, surgical assistance, and outcome assessment.

## 1. Introduction

Osteoarthritis is a degenerative disease that involves destruction of joint articular cartilage, mainly a breakdown of type II collagen and proteoglycans. This degradation, as well as adverse remodeling of subchondral bone, is mediated and propagated by inflammatory cytokines and other mediators [1-4]. Risk factors for the development and diagnosis of osteoarthritis include aging, genetics, obesity, vitamin D deficiency, and trauma [5-9]. In 2020, the estimated worldwide incidence of osteoarthritis of the knee, specifically, was 86.7 million people [10].

There are several therapeutic approaches in the treatment of osteoarthritis [11]. Surgical interventions, namely arthroplasties, are a common and effective treatment for knee osteoarthritis to reduce pain and improve function [12-15]. Heckmann et. al. [16] estimates that 645,852 elective knee arthroplasties were performed in the United States from 2017-2019 and this number is projected to reach 3.48 million annually by 2030 [17]. According to the American Joint Replacement Registry 2023 Annual Report, the mean age of 1.8 million people who underwent knee arthroplasties from 2012-2022 was 67.4 years [18]. Both the diagnosis of knee osteoarthritis and intervention via arthroplasty are more common in females [2,19].

The last 30 years has seen considerable expansion in the use of technology in Orthopedic Surgery and knee arthroplasties, primarily related to robotics and computer-assisted surgeries [20]. Artificial intelligence (AI) is a technology that is commonly used along-side computer-assisted surgery but also has solo applications in the field of joint reconstructive surgery. AI uses pattern recognition and algorithms to provide classifications, predictions, and solutions to tasks with a goal of greater efficiency than humans. It is an umbrella term that encompasses subsets such as machine learning (ML) and deep learning (DL). Though knee arthroplasty is an increasingly common procedure, it is associated with negative outcomes such as patient dissatisfaction, surgical complications, limited functionality, and revision surgery [21]. AI in knee arthroplasty has the potential to streamline operations, limit negative outcomes and improve patient quality of life as the number of patients undergoing this procedure continues to grow.

Much of current literature reviews robotic-assisted knee arthroplasty or the use of AI on specific aspects of the procedure from analyzing diagnostic imaging to predicting outcomes. Moreover, existing reviews discuss AI and ML but tend to not mention the additional subset of DL. The objective of this article is to provide an overview of current research on the application of AI and its subsets in each stage of knee arthroplasties.

## 2. Methods

An initial search on PubMed revealed ML and DL as common MeSH terms and keywords used alongside AI in knee arthroplasties and were included in this review. The following search terms and Boolean operators were used to search PubMed for studies relevant to the current application of AI in knee arthroplasties: knee arthroplasty, knee replacement, total knee arthroplasty, artificial intelligence, machine learning, and deep learning. We did not use the terms review, systematic review, meta-analysis, and editorial, and accordingly did not gather information from such articles. PubMed filters were used to ensure that only studies published in English from 2019-2024 were included. Research involving AI in all types of knee arthroplasty procedures, such as partial knee arthroplasties, total knee arthroplasties (TKA), and revisions, were included. Articles were manually reviewed and commentaries, bibliometric studies, letters to the editor, and papers irrelevant to the research question were excluded. Articles discussing computer-assisted surgery, robotic-assisted surgery, and the use of statistical analyses such as linear regression without explicit statement of use of ML, DL, or AI in knee arthroplasty were also excluded. Data regarding the article title and year published were extracted and organized via spreadsheet. Additionally, the content of each article was analyzed for the levels of evidence, methods, and key findings. Studies were categorized as AI relating to preoperative, perioperative or postoperative knee arthroplasty. Preoperative articles were further specified into predicting and assessing the need for knee arthroplasty or pre-operative patient education and decision making. Postoperative articles were further specified into predicting and assessing adverse outcomes or other post-operative outcomes. A representative selection of articles with the highest level of evidence in each of the three categories has been summarized and presented in Table 1-3.

## 3. Results

**Figure 1:**
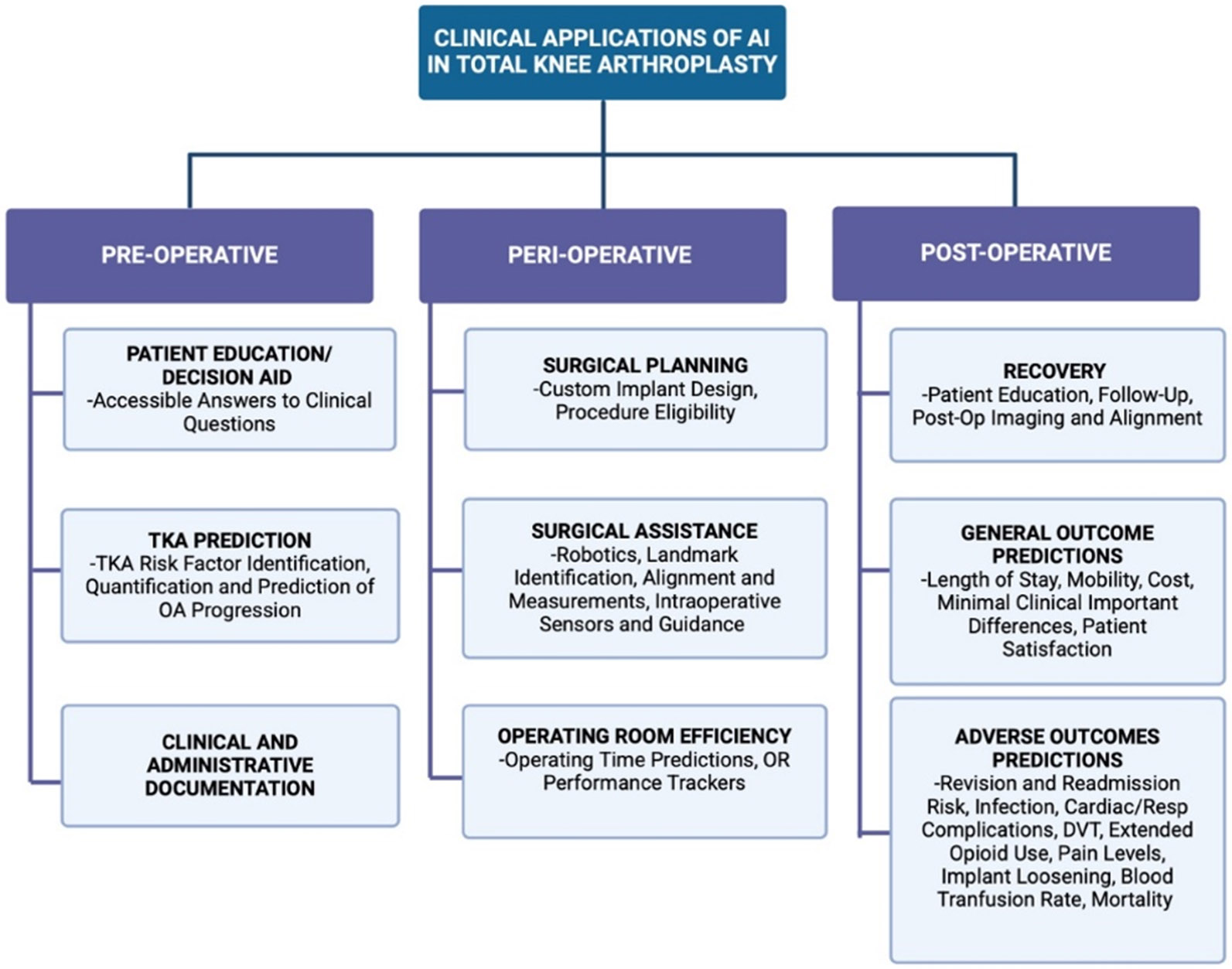
Pre-operative, peri-operative, and post-operative clinical applications of AI and involved procedures and steps in both primary TKA and revision TKA. DVT, deep venous thrombosis.

A search in the last 5 years of the defined terms in PubMed produced 423 articles published in English. 182 were deemed relevant based on the research question of this review and pre-defined inclusion and exclusion criteria. 35/182 (19.23%) studies examined the use of AI prior to knee arthroplasty procedure with 21 of these articles discussing AI in predicting the incidence and risk of knee arthroplasty and 14 analyzing AI as a tool for pre-operative decision making and patient education. 42/182 (23.08%) of articles discussed perioperative uses of AI, including how AI can directly impact the execution of surgical procedures (Figure 1). Lastly, 105/182 (57.69%) of research regarding AI in knee arthroplasties considers the prediction of post-surgical outcomes with 63 specifically evaluating risk assessments and the potential for adverse effects.

## 4. Discussion

This review identified 182 research studies published from 2019-2024 that analyze the use of AI, ML, and DL in the preoperative, perioperative, or postoperative phases of knee arthroplasties. The decision to cover explicit uses of AI and its subsets in knee arthroplasty, especially with regards to computer and robotic assisted surgeries, is a strength of this review. This provides a perspective specific to technology that can gather knowledge from patterns of data to complete tasks and make predictions and can be used for more than just intraoperative robotic assistance.

The use of AI and its subsets in the preoperative stage has potential implications for the accessibility of information and provides the potential to standardize patient education and understanding of the knee arthroplasty procedure. It is of note that of the 12/14 studies included in this review which analyze AI as a tool for pre-operative decision making and patient education were published in 2023-2024. This highlights a more recent direction of research focused on investigating patient interaction with AI, particularly the AI large language model ChatGPT. However, the emergence of AI and its active role in guided decision making raises multiple ethical and privacy concerns [37,38]. Patient and provider mistrust of AI may be further explained by the term ‘AI Black Box,’ which refers to the lack of transparency and explanation for output models that presents a difficulty for humans to interpret and explain [39].

Commonly, radiographic analysis is used to diagnose the severity of osteoarthritis, which is one of the factors used to determine the need for treatment via knee arthroplasty [40,41]. Manual analysis of diagnostic imaging leaves room for potential human error. Therefore, AI can provide a more objective method for diagnosing severe osteoarthritis and using patient factors to identify those at risk for future knee arthroplasty to offer improved quality of life to patients affected by osteoarthritis.

The critical review of multiple studies identified perioperative and intraoperative use of AI as the second greatest research field of AI application in knee arthroplasty with 42/182 (22.08%) of articles. This highlights the direct effect that AI can have on surgical planning and implementation. Moreover, AI has intraoperative uses outside of robotic-assisted surgery, primarily with regards to determining proper implant sizes and soft tissue balancing.

Another strength of this review is the quantification of the scope of AI use in the stages of all types of knee arthroplasty procedures, including partial, total, and revision arthroplasties. With the growing projected prevalence of knee arthroplasties also comes the projected increase in arthroplasty revision [17]. Revision knee arthroplasties are commonly due to factors such as component loosening or periprosthetic joint infection [42,43]. In addition to revisions, primary knee arthroplasties are also subject to adverse outcomes such as cardiovascular and respiratory complications and bleeding [44]. This findings in this article indicate that the majority of AI and knee arthroplasty -related research (105/182 articles) consists of using AI to perform risk assessments and predict overall treatment outcomes. This finding supports the necessity to improve discharge planning, patient reported outcome measures, and additional factors to advance outcomes associated with knee arthroplasty.

## 5. Limitations and Future Directions

This information in this article is limited in the fact that grouping of articles was performed by a single reviewer, which could present a perceived bias and subjective categorization despite objective evaluation of each published finding. This study also does not discuss an in-depth analysis of the quality of articles included. An overview of the types of studies and level of investigation of AI and knee arthroplasty related research as well as the data used to train AI, ML and DL programs (electronic medical records, diagnostic imaging) would be of interest. Moreover, future investigation into the scope research comparing the efficacy between types of AI and statistical analysis such as logistic regression could be beneficial to the field.

Ultimately, AI is an emerging resource and has various applications through every stage of knee arthroplasties. Research is currently being conducted to assess the use of AI and its subsets ML and DL to improve patient experience, clinical practice, and surgical assistance.

## Figures and Tables

**Table 1:** The purpose and the key findings from pre-operative studies.

Type of Study	Purpose	Key Findings	Reference
Randomized Controlled Trial	This randomized controlled non-inferiority trial investigates the efficacy and cost-effectiveness of an AI-based system in supporting patients’ decision to undergo TKA, compared to standard pre-operative education.	The study protocol predicts that there will be no significant difference in clinical outcomes between the AI-supported group and the control group receiving standard pre-operative education for TKA.	Kastrup et al. [22]
Randomized Controlled Trial	This randomized controlled trial compares an AI-enabled patient decision aid to standard educational material for patients with advanced knee osteoarthritis considering TKA.	Patients who received the AI-enabled, personalized decision aid demonstrated significantly better decision quality, higher patient satisfaction, greater collaboration in shared decision-making, and improved functional outcomes compared to those in the control group, who only received standard educational material.	Jayakumar et al. [23]
Randomized Controlled Trial	This randomized controlled trial studies the ability of a tool that uses ML algorithms to impact patient decisions and willingness to undergo TKA, based on individualized predicted surgical outcomes.	The protocol for this pragmatic trial includes patients diagnosed with knee osteoarthritis who are considering TKA. Participants are randomized to either receive the prognostic tool or usual care. The study also explores the optimal timing for tool use in the patient decision-making process and examines its influence on decision quality.	Zhou et al. [24]
Analysis of Prospective Datasets	This study utilizes data from prospective cohort studies to create and assess six ML models and their ability to predict the need for TKA in 2 to 5 years.	The Gradient Boosting Machine model proved to be a strong predictive tool for TKA, particularly at 2 years, with slightly reduced accuracy at 5 years. Radiographic-derived features, questionnaires, and patient education level were identified as significant predictive features.	Mahmoud et al. [25]
Secondary Analysis of a Prospective Study	This study analyzes quadriceps muscle atrophy and intramuscular adipose tissue (intra-MAT) in patients with osteoarthritis as potential predictors of TKA, using DL segmentation models and MRI scans.	Patients with knee osteoarthritis were found to have smaller quadriceps cross-sectional areas and larger amounts of intramuscular adipose tissue compared to patients without osteoarthritis. The study establishes that increased intramuscular adipose tissue is significantly linked to heightened likelihood of TKA and worsened osteoarthritis symptoms. Additionally, quadriceps atrophy over time is negatively associated with the risk of TKA and worsening osteoarthritis symptoms.	Mohajer et al. [26]

**Table 2:** The purpose and the key findings from the peri-operative studies.

Type of Study	Purpose	Key Findings	Reference
Experimental Study	This experimental study validates an intraoperative sensor that uses AI to predict load and location of forces to assist in soft tissue balancing during TKA.	The AI-based intraoperative sensor achieved significant accuracy and precision in detecting load values, with an average of 83.41% for load detection and 84.63% for force location estimates.	Al-Nasser et al. [27]
Experimental Study	This experimental study utilizes DL to analyze MRIs of thigh muscles and use this data to inform the design process of TKA prosthetics.	AI-generated quantitative and qualitative analyses of thigh muscles from MRI data were used to develop prosthetics for TKA to improve the customization and control of the devices.	Arunachalam et al. [28]
Experimental Study	This experimental study validates the use of ML algorithms to generate 3D models from 2D diagnostic imaging to assist with implant sizing and alignment for TKA surgery.	The AI-generated 3D models measured femoral and tibial landmarks with sub-millimeter accuracy, which are important for precise implant sizing and alignment during TKA.	Factor et al. [29]
Experimental Study	This experimental proof-of-concept study utilizes DL to guide navigation sensors for bone segmentation during TKA.	The study demonstrates the feasibility of AI-guided segmentation for contactless registration of bone surfaces, using RGB (red, green and blue wavelengths) and depth cameras, during TKA. The experimental results confirm that this system can provide real-time guidance to surgeons, reducing the need for traditional invasive markers.	Rodrigues et al. [30]
Cadaveric Study	This cadaveric study compares knee measurements obtained from AI-generated 3D reconstructions of 2D X-ray diagnostic images to those determined from CTs and manually by Orthopedic Surgeons.	AI-generated 3D reconstructions of 2D CT and X-ray diagnostic images provided accurate knee landmarks and measurements to be used for implant placement during TKA. All but one AI-based measurement produced a mean absolute error < 2mm compared to standard manual measurements.	Fernandes et al. [31]

**Table 3:** The purpose and the key findings from the post-operative studies.

Type of Study	Purpose	Key Findings	Reference
Randomized Controlled Trial	This randomized controlled trial examined the impact of electronic Patient Reported Outcome Measure (ePROM) monitoring at 1-, 3-, 6- and 12-months post arthroplasty compared to standard 12-month PROM assessments. Using ML, the study explored variations in treatment effects across different patient subgroups.	It was found the ePROM monitoring was particularly beneficial for certain groups, such as women over 65 years of age, those with blood pressure issues, and those not employed post-surgery. Knee arthroplasty patients who were not obese and had discussions about their PROM scores with their doctors also showed more significant improvements. The intervention highlighted the importance of targeting specific subgroups to optimize post-surgical outcomes.	Langenberger et al. [32]
Prospective Cohort Study	This prospective cohort study compares several ML models for predicting post-operative outcomes after TKA using electronic medical record data.	The ML models demonstrated the highest accuracy in predicting the subscales of the Knee Injury and Osteoarthritis Outcome Score (KOOS) – activities of daily living, pain, symptoms, and quality of life – compared to broader measures like the KOOS Total and KOOS Jr. These models were validated for their accuracy and efficiency, with varying number of inputs required for different subscales.	Harris et al. [33]
Prospective Cohort Study	This prospective cohort study validates the use of a ML-based wearable sensor to passively and continuously monitor mobility, knee reported outcome measure, and home exercise program compliance after TKA. The study also tracked PROMs and opioid use through weekly surveys on a mobile app.	Patients reported the system as “motivating” and “engaging” and the continuous data collection provided real-time feedback on recovery.	Ramkumar et al. [34]
Pilot Cohort Study	This pilot cohort study identifies the most appropriate bedside screening tools for assessing pain sensitization in chronic osteoarthritis patients post-TKA using ML.	ML models identified pressure pain sensitivity, mechanical pinprick pain sensitivity over the most affected knee, and extra segmental pressure pain sensitivity as the best tools for detecting sensitization in these patients.	Sachau et al. [35]
Prospective Analysis	This prospective study evaluates the accuracy and appropriateness of ChatGPT (an AI-based large language model) compared to arthroplasty-trained nurses in answering common postoperative questions for patients who have undergone TKA.	Both ChatGPT and the nurses provided appropriate answers to frequently asked patient questions, as determined by fellowship-trained surgeons. Additionally, over 90% of patient’s surveyed were uncertain about trusting AI to answer their postoperative questions.	Bains et al. [36]

## Key Points

- Artificial intelligence (AI), including machine learning (ML) and deep learning (DL), has applications in the preoperative, perioperative, and postoperative stages of knee arthroplasties, improving diagnostics, surgical precision, and patient outcomes.
- AI is increasingly used for patient education and decision-making before surgery. Recent studies (2023-2024) focus on the role of AI in assisting patients with surgical decisions, including using ChatGPT as an informational tool.
- The use of AI in perioperative settings extends beyond robotic-assisted surgery, significantly enhancing implant positioning, soft tissue balancing, and intraoperative navigation.
- AI applications in knee arthroplasty are rapidly expanding, with over 57% of recent studies focusing on postoperative outcome prediction and risk assessment.
- Despite growing integration of AI in knee arthroplasty, concerns over patient trust, data privacy, and ethical implications remain key barriers to widespread clinical adoption.
- While AI applications in knee arthroplasty are expanding, further research is needed to compare different AI models, validate their efficacy against traditional statistical approaches, and establish standardized implementation protocols.
